# Randomized phase II study of daily and alternate-day administration of S-1 for advanced gastric cancer (JFMC43-1003)

**DOI:** 10.1007/s10147-017-1157-3

**Published:** 2017-06-30

**Authors:** Hiroaki Tanaka, Mitsuro Kanda, Satoshi Morita, Masataka Taguri, Kazuhiro Nishikawa, Mitsuo Shimada, Kazuya Muguruma, Keisuke Koeda, Masazumi Takahashi, Mikihito Nakamori, Hiroyuki Konno, Akihito Tsuji, Yoshinori Hosoya, Tetsuhiko Shirasaka, Susumu Yamamitsu, Michio Sowa, Masaki Kitajima, Masazumi Okajima, Michiya Kobayashi, Junichi Sakamoto, Shigetoyo Saji, Kosei Hirakawa

**Affiliations:** 1 Department of Surgical Oncology, Osaka City University Graduate School of Medicine, 1-4-3 Asahimachi, Abenoku, Osaka, 545-8585 Japan; 2 Department of Gastroenterological Surgery (Surgery II), Nagoya University Graduate School of Medicine, Nagoya, Japan; 3 Department of Biomedical Statistics and Bioinformatics, Kyoto University Graduate School of Medicine, Kyoto, Japan; 4 Department of Biostatistics, Yokohama City University Graduate School of Medicine, Yokohama, Japan; 5 Department of Surgery, Osaka National Hospital, Osaka, Japan; 6 Department of Surgery, Tokushima University, Tokushima, Japan; 7 Department of Surgery, Iwate Medical University, Morioka, Japan; 8 Department of Gastrointestinal Surgery, Yokohama Municipal Citizen’s Hospital, Yokohama, Japan; 9 Second Department of Surgery, Wakayama Medical University, Wakayama, Japan; 10 Hamamatsu University School of Medicine, Hamamatsu, Japan; 11 Clinical Oncology, Faculty of Medicine, Kagawa University, Takamatsu, Japan; 12 Department of Gastrointestinal Surgery, Jichi Medical University, Shimotsuke, Japan; 13 Tokushima University, Kitasato University, Tokushima, Japan; 14 Sapporo Tsukisamu Hospital, Sapporo, Japan; 15 History of Yukioka School of Allied Health Professions, Osaka, Japan; 16 International University of Health and Welfare, Otawara, Japan; 17 Department of Surgery, Hiroshima City Hiroshima Citizens Hospital, Hiroshima, Japan; 18 Cancer Treatment Center, Kochi Medical School Hospital, Kochi, Japan; 19 Japanese Foundation for Multidisciplinary Treatment of Cancer, Tokyo, Japan; 20 Osaka City University Hospital, Osaka, Japan

**Keywords:** S-1, Chemotherapy, Gastric cancer, Randomized phase II study

## Abstract

**Purpose:**

Although S-1 based chemotherapy for patients with advanced gastric cancer has generally been accepted in Japan, discontinuations of treatment have been reported due to grade 3 or more adverse events. The present randomized phase II study was conducted to test whether alternate-day administration of S-1 would be comparably efficient and reduce adverse events compared with conventional daily administration in the first-line chemotherapy for advanced gastric cancer.

**Methods:**

132 patients with advanced gastric cancer were randomly assigned to 1:2 ratios to receive treatment with daily at a standard dose of 80 mg/m^2^/day or alternate-day administration group received S-1 on 4 days a week. The primary end point was progression-free survival (PFS), and the secondary end points were safety, overall survival, time to treatment failure (TTF), disease control rate, and response rate.

**Results:**

The 6-month PFS rate of the alternate-day administration group was 20.9% and failed to show significant difference from the pre-specified threshold at 15% (*p* = 0.117), whereas that of the daily administration group was 39.1% and significantly higher than the threshold (*p* = 0.001). The hazard ratio of the alternate-day administration group compared with the daily administration group was 1.753 (95% confidence interval (CI) 1.15–2.68, *p* = 0.010). With regard to OS, the hazard ratio of the alternate-day administration group compared with the daily administration group was 1.487 (95% CI 0.97–2.29, *p* = 0.072). The median TTF were 4.2 and 2.8 months in the daily and alternate-day administration group, respectively (*p* = 0.007).

**Conclusion:**

The alternate-day administration of S-1 was not recommended as the first-line therapy for patients with advanced gastric cancer.

## Introduction

Gastric cancer is the second commonest cause of cancer-related death in the world [1]. Since 1999, S-1, an oral anticancer drug, has been positioned as a key drug for first-line chemotherapy of advanced gastric cancer in view of its effectiveness in Japan [2]. S-1 is an oral combined form of tegafur (pro drug of 5-fluorouracil; 5-FU) and two biochemical modulators, gimeracil and oteracil [3]. Gimeracil is a potent reversible inhibitor of 5-FU degradation; and oteracil is an inhibitor of the enzyme that catalyzes the phosphorylation of 5-FU and reduces gastrointestinal toxicity of 5-FU. Two previous phase II studies showed that the response rate of S-1 alone for advanced gastric cancer was 44 and 49%, respectively, and the incidence of grade 3 or more adverse events was 20% [4, 5]. As shown in a previous phase III clinical trial evaluating non-inferiority of S-1 compared with fluorouracil in patients with metastatic gastric cancer, S-1 has some advantages over continuous infusion of fluorouracil in view of the convenience of an oral administration [6].

Although the toxic effects of S-1 have been reported to be acceptable, the frequency of grade 3 or higher adverse events was 24.7% in patients assigned to S-1 alone in the SPIRITS trial and 20% in phase II trials [5, 7]. Additionally, long-lasting treatment is very important to demonstrate the effect of S-1 on prognosis. For example, the median survival time (MST) of peritoneal cytology-positive patients that could take S-1 for more than 1 year was 850 days [8]. It is urgent to continue to examine a regimen with fewer side effects and longer treatment duration to improve the outcomes of advanced gastric cancer.

Changing the method of administration, including alternate-day administration, has been examined as an example of reducing severe adverse effects. Several clinical trials to verify the clinical effect of alternate-day administration of S-1 are currently in progress for other carcinomas, such as pancreatic cancer and colorectal cancer [9]. A phase II study for locally advanced and metastatic pancreatic cancer by Yamaue et al. showed that the incidence of grade 3 or higher hematological toxicities were 4.2%, and progression-free survival (PFS) was 5.5 months in patients treated with alternate-day administration [10]. With regard to gastric cancer, a retrospective study reported that, compared with daily administration, alternate-day administration of S-1 reduced adverse effects without sacrificing clinical outcomes and showed longer average treatment duration [11]. However, no prospective study has investigated the effectiveness of alternate-day administration of S-1 for advanced gastric cancer so far.

The aim of this randomized, phase II study was to examine the hypothesis that alternate-day administration of S-1 can show equivalent clinical efficacy while reducing adverse events compared with standard daily administration regimen in the first-line chemotherapy for advanced gastric cancer.

## Patients and methods

### Study design

The Japanese Foundation for Multidisciplinary Treatment of Cancer (JFMC) 43-1003 study was a multicenter, prospective, randomized, open-label, phase II trial. Patients were randomly assigned in 1:2 ratio to receive treatment with daily or alternate-day administration of S-1.

### Patients

The eligibility criteria for patients were as follows: histologically proven gastric adenocarcinoma; unresectable or recurrent disease (only peritoneal cytology-positive cases were excluded); a measurable lesion confirmed 28 days prior to enrollment; no prior treatment (more than 180 days after the end of postoperative adjuvant chemotherapy); preserved organ functions [white blood cell count of 3.0–12.0 × 10^3^/mm^3^; number of neutrophils ≥2.0 × 10^3^/mm^3^; number of platelets ≥10.0 × 10^3^/mm^3^; hemoglobin ≥8.0 g/dl; total bilirubin ≤1.5 mg/dl; AST/ALT ≤100 IU/l; creatinine clearance (CCr) ≥50 ml/min]; the Eastern Cooperative Oncology Group performance status 0–2; survival expectation ≥3 months; age ≥20 years; possible oral intake; no abnormal findings on electrocardiogram within 28 days before entry; and that patients provided written, informed consent to participate. Patients with severe peritoneal ascites or brain metastasis were excluded. After stratification according to institution, performance status (0 or 1 or 2), and unresectable pattern (metastatic or recurrence), patients were randomly assigned to receive either daily or alternate-day administration of S-1.

### Treatment

The daily administration group received S-1 orally twice daily for the first 4 weeks of a 6-week cycle. The alternate-day administration group received S-1 twice in 4 days (Monday, Wednesday, Friday and Sunday) a week. S-1 was then administered according to the schedule for the alternate day regimen without changing the day of week for a 6-week cycle. The dose of S-1 administered per day was based on the patient’s body surface area as follows: <1.25 m^2^, 80 mg; 1.25–1.50 m^2^, 100 mg; >1.5 m^2^, 120 mg. Reduction of doses was configured in two stages according to CCr or laboratory data at the start of each cycle. Reduced doses per day at the first stage and second stage were as follows: <1.25 m^2^, 50 and 0 mg; 1.25–1.50 m^2^, 80 and 50 mg; >1.5 m^2^, 100 and 80 mg. The criteria of dose reduction were as follows: white blood cell count <1.0 × 10^3^/mm^3^; number of neutrophils <5.0 × 10^2^/mm^3^; number of platelets <2.5 × 10^3^/mm^3^; grade 3 or higher diarrhea and grade 3 or higher stomatitis. The criteria to continue administration were as follows: white blood cell count of 2.0–12.0 × 10^3^/mm^3^; number of neutrophils ≥1.0 × 10^3^/mm^3^; number of platelets ≥7.5 × 10^3^/mm^3^; CCr ≥50 ml/min; diarrhea <grade 1; appetite loss <grade 2; nausea <grade 2. If administration continuity criteria were not met at all points in the course, administration of S-1 was discontinued. Within 7 days after discontinuation, S-1 administration resumed if administration continuation criteria were met. Schedule change of 2 weeks on and 1 week off etc. was not permitted. Treatment of both groups was continued until one of the following occurred: progressive disease, treatment was not resumed even after 28 days from the last administration, administration difficulty due to adverse effects, or decision to stop treatment at the discretion of the treating physician.

### Study parameters

The subject of analysis in this study was full analysis set (FAS), that is, eligible cases in which S-1 was administered even once. The primary end point was progression-free survival (PFS), and the secondary end points were safety, overall survival (OS), time to treatment failure (TTF), disease control rate (DCR), and response rate (RR). Tumors were measured every 6 weeks and assessed according to the Response Evaluation Criteria in Solid Tumor version 1.1 (RECIST). Based on the evaluation value judged according to RECIST, the best overall effect was taken as a numerator, the response rate with the measurable lesion as a denominator, and the DCR calculated for each treatment group. Adverse events were according to the Common Terminology Criteria for Adverse Events version 4.02 (CTCAE v4.02).

### Statistical analysis

In this trial, sample size was determined based on a study design in which each treatment group (daily administration group; alternate-day administration group) is evaluated as to whether an effect that at least exceeds the threshold is exhibited. We referred data of a randomized phase III clinical trial, the SPIRITS trial, that demonstrated the survival benefit of S-1 plus cisplatin compared to S-1 monotherapy in patients with advanced gastric cancer [7]. In the SPIRITS trial, 6-month progression-free survival in the S-1 daily administration group was 26%. With a significance level of 5% (one-tailed), power of 80%, a 6-month progression-free survival threshold of 15%, and an expected 6-month progression-free survival of 26%, the necessary sample size in this trial was calculated as 76 (one-tailed). There was a concern in delayed recruitment due to a limited number of participants because S-1 monotherapy was no longer the standard treatment for advanced gastric cancer after the SPIRITS trial. Therefore, we prescribed in the study protocol that integrated data analysis of the JFMC43-1003 (the present study) and 40 patients from the SPIRITS trial if no significant differences in PFS after adjustments with propensity score weighting were found between the two cohorts for the daily administration group. Eventually, 40 for the daily administration and 80 for the alternate-day administration groups were set as the target sample sizes. An interim analysis of the incidence of adverse events and 6-week progressive disease rate was conducted about 2 months after the enrollment of 20 patients in daily group and 40 patients in alternate-day group.

To compare quantitative data, we used the Wilcoxon signed-rank test for within-patient comparisons. The Kaplan–Meier method was used to calculate survival curves, and the stratified log-rank test was used for comparisons. We considered *p* < 0.05 statistically significant.

### Ethics statement

This study was approved by the ethics committee of participating institutions and carried out according to the Declaration of Helsinki.

## Results

### Patient characteristics

**Fig. 1:**
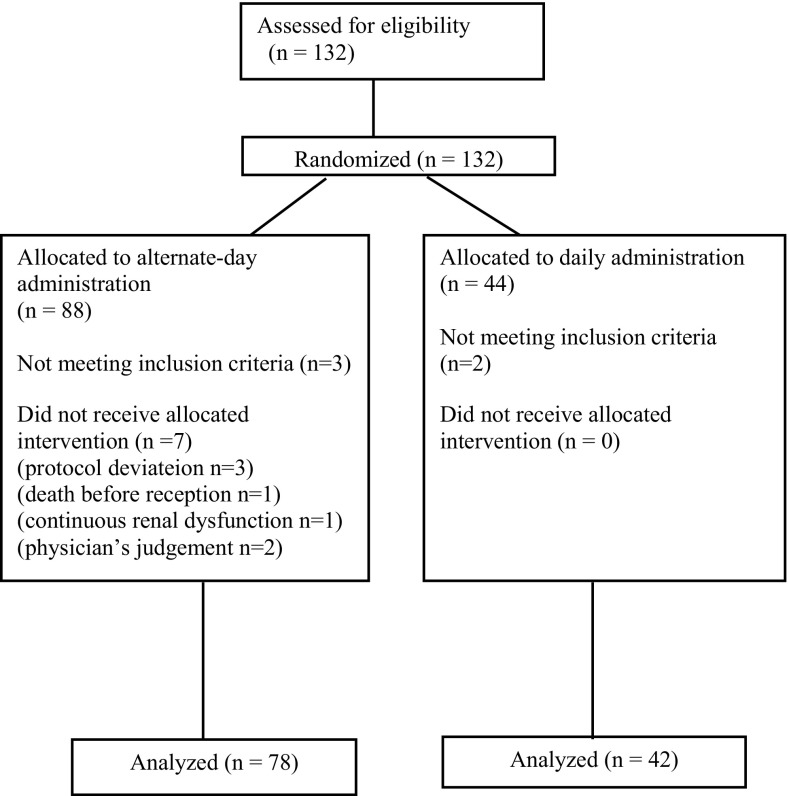
CONSORT diagram

During the period from December 2010 to November 2012, 132 patients who met the inclusion criteria were randomized in this study from 21 different institutions (Fig. 1). At enrolment, five patients did not meet the eligibility criteria: one was excluded due to a different diagnosis, one was due to a psychological disorder, and the others did not have measurable lesions. The full analysis set comprised of 120 patients (42 in the daily administration group and 78 in the alternate-day administration group). The baseline characteristics of the patients are summarized (Table 1). There were some differences in patients’ characteristics between the groups. Body surface area was larger in the alternate-day administration group than in the daily administration group, and the alternate-day administration group had a tendency to have more Type 4 cancer and overall location of the tumor.

**Table 1:** Patients’ baseline characteristics

	The daily administration group	The alternate-day administration group	*p* value
	*n* = 44	*n* = 88	
Sex			
Men	26	59	0.368
Women	18	29	
Age			
Median (range)	73 (43–89)	75 (53–87)	0.330
Height			
Median (range)	155.3 (143.5–171.0)	160.0 (132.6–176)	0.029
Body weight			
Median (range)	49.9 (33.1–82.0)	54.2 (30.0–80.0)	0.045
PS			
PS0	33	67	1.000
PS1	9	18	
PS2	2	3	
Body surface area			
Median (range)	1.41 (1.13–1.90)	1.52 (1.10–1.91)	0.030
Occupation site			
E	2	1	0.049
U	16	23	
M	14	18	
L	12	42	
D	0	0	
Overall	0	4	
Histology			
Differentiated types	21	45	0.556
Undifferentiated types	21	41	
Others	2	2	
Macroscopic type			
Type 1	3	1	0.059
Type 2	4	21	
Type 3	29	41	
Type 4	6	19	
Type 5	1	4	
Gastrectomy			
No	23	42	0.624
Yes	21	46	
Eligibility			
Eligible	42	85	0.092
Disqualification	2	3	

### Treatment

**Fig. 2:**
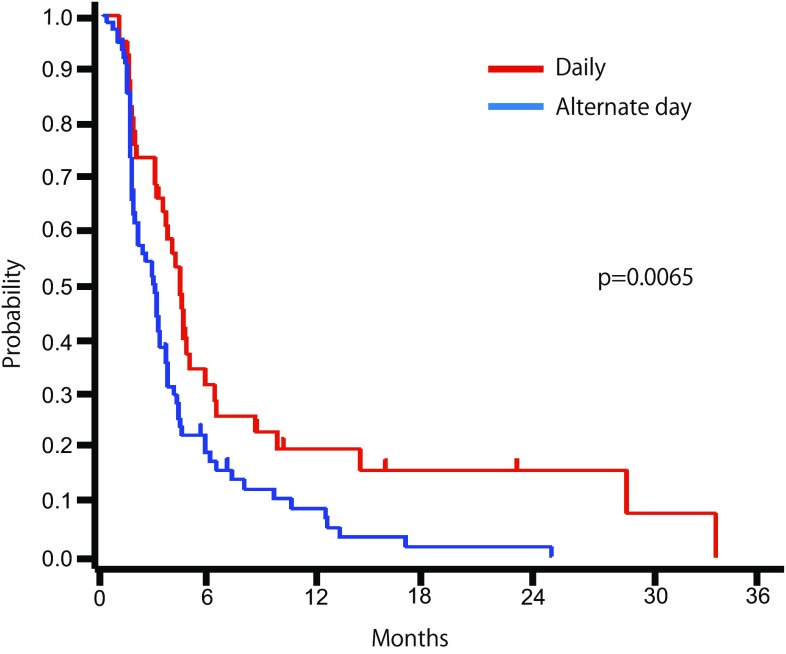
Time to Treatment failure

The summary of treatment is shown in Table 2. The median number of courses of administration was 3.0 and 2.0 in the daily and alternate-day administration groups, respectively. The median total dose of S-1 was 6610 and 3140 mg in the daily and alternate-day administration groups, respectively, and the difference was statistically significant. The rate of dose reduction was similar in both groups. The most common reason for withdrawal of treatment was progressive disease. The total number of administration days was significantly shorter in the alternate-day administration group than in the daily administration group. The percent of patients who discontinued treatment within 6 weeks was 34.6% in the alternate-day administration group and 19% in the daily administration group. With regard to the RR and DCR, patients assigned to the alternate-day administration group had more progressive disease compared with those assigned to the daily administration group (Table 2). The median time to treatment failure were 4.2 months (95% CI 2.86–4.70) in the daily administration group and 2.8 months (95% CI 1.64–3.06) in the alternate-day administration group (*p* = 0.007) (Fig. 2). The common adverse events are summarized in Table 3. The most common hematological toxicity was anemia (73.8% in the daily administration group, 71.1% in the alternate-day administration group) (Table 3). Grade 3 or higher adverse events included anorexia (10%), anemia (7%), neutropenia (5%), and fatigue (5%) in the daily administration group and anemia (10%) and anorexia (5%) in the alternate-day administration group.

**Table 2:** Summary of treatment

	The daily administration group	The alternate-day administration group	*p* value
	*n* = 42	*n* = 78	
Administration week (course/6 week)			
Average ± standard deviation	4.5 ± 5.16	2.9 ± 2.91	
Total dosage (mg)			
Median (range)	6610.0 (400.0–67200.0)	3140.0 (120.0–42200.0)	0.0056
Administration date			
Median (range)	69.0 (4.0–672.0)	31.5 (1.0–422.0)	0.0007
Response rate			
CR	0	0	0.0152
PR	13	10	
SD	18	33	
PD	9	29	
Disease control ratio	31/42 (73.8%)	43/75 (57.3%)^a^	

^a^Except 3 patients with missing values

**Table 3:** Adverse effects by the treatment groups

Event	The daily administration group (*n* = 42)						The alternate-day administration group (*n* = 78)					
	Grade 1 (%)	Grade 2 (%)	Grade 3 (%)	Grade 4 (%)	Grade 5 (%)	≥Grage3 (%)	Grade 1 (%)	Grade 2 (%)	Grade 3 (%)	Grade 4 (%)	Grade 5 (%)	≥Grade 3 (%)
Anemia (Hb)	8 (19)	20 (48)	3 (7)	0 (0)	0 (0)	3 (7)	29 (37)	18 (23)	6 (8)	2 (3)	0 (0)	8 (10)
Leukopenia	12 (29)	4 (10)	0 (0)	1 (2)	0 (0)	1 (2)	6 (8)	5 (6)	0 (0)	0 (0)	0 (0)	0 (0)
Neutropenia	10 (24)	4 (10)	1 (2)	1 (2)	0 (0)	2 (5)	5 (6)	5 (6)	2 (3)	0 (0)	0 (0)	2 (3)
Thrombocytopenia	8 (19)	1 (2)	0 (0)	1 (2)	0 (0)	1 (2)	12 (15)	1 (1)	0 (0)	0 (0)	0 (0)	0 (0)
Total bilirubin increase	9 (21)	4 (10)	0 (0)	0 (0)	0 (0)	0 (0)	9 (12)	6 (8)	1 (1)	0 (0)	0 (0)	1 (1)
AST increase	7 (17)	2 (5)	1 (2)	0 (0)	0 (0)	1 (2)	13 (17)	3 (4)	1 (1)	0 (0)	0 (0)	1 (1)
ALT increase	4 (10)	2 (5)	0 (0)	0 (0)	0 (0)	0 (0)	11 (14)	1 (1)	1 (1)	0 (0)	0 (0)	1 (1)
Anorexia	13 (31)	9 (21)	4 (10)	0 (0)	0 (0)	4 (10)	17 (22)	13 (17)	4 (5)	0 (0)	0 (0)	4 (5)
Nausea	7 (17)	5 (12)	1 (2)	0 (0)	0 (0)	1 (2)	12 (15)	10 (13)	3 (4)	0 (0)	0 (0)	3 (4)
Vomiting	3 (7)	1 (2)	1 (2)	0 (0)	0 (0)	1 (2)	7 (9)	2 (3)	1 (1)	0 (0)	0 (0)	1 (1)
Oral mucositis	6 (14)	3 (7)	0 (0)	0 (0)	0 (0)	0 (0)	0 (0)	0 (0)	0 (0)	0 (0)	0 (0)	0 (0)
Diarrhea	6 (14)	2 (5)	0 (0)	0 (0)	0 (0)	0 (0)	7 (9)	1 (1)	0 (0)	0 (0)	0 (0)	0 (0)
Fatigue	11 (26)	5 (12)	2 (5)	0 (0)	0 (0)	2 (5)	12 (15)	7 (9)	3 (4)	0 (0)	0 (0)	3 (4)
Rash	5 (12)	1 (2)	0 (0)	0 (0)	0 (0)	0 (0)	3 (4)	0 (0)	0 (0)	0 (0)	0 (0)	0 (0)

### Relinquishment of data integration

Since the study protocol prescribed the data integration with the provision that there were no significant differences in PFS between the daily administration group of the JFMC43-1003 and SPIRITS cohorts, we compared the PFS after propensity score weighting. We found a significant difference in PFS between the two cohorts (hazard ratio of the SPIRITS cohort 1.579, 95% CI 1.03–2.42, *p* = 0.036).

### Survival

**Fig. 3:**
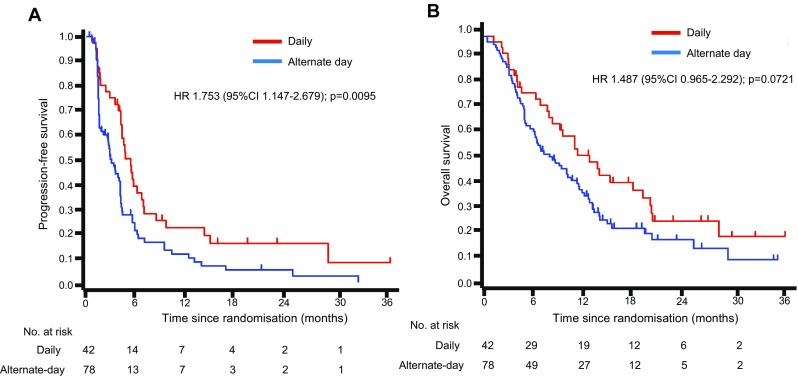
Kaplan–Meier curves of progression-free survival progression-free survival (**a**) and overall survival (**b**). *HR* hazard ratio

The median PFS was 5.32 (95% CI 4.13–6.60) in daily administration group and 3.05 (95% CI 1.83–4.10) in the alternate-day administration group (Fig. 3a). The 6-month PFS rate of the alternate-day group was 20.9% and failed to show significant difference from the pre-specified threshold at 15% (*p* = 0.117), whereas that of the daily group was 39.1% and significantly higher than the threshold (*p* = 0.001). The hazard ratio of the alternate-day administration group compared with the daily administration group was 1.753 (95% CI 1.15–2.68, *p* = 0.010). The median OS was 12.61 months (95% CI 8.11–19.02) in the daily administration group and 8.14 months (95% CI 6.01–11.2) in the alternate-day administration group. With regard to OS, the hazard ratio of the daily administration group compared with the alternate-day administration group was 1.487 (95% CI 0.97–2.29, *p* = 0.072) (Fig. 3b).

## Discussion

To our best knowledge this study is the first clinical trial to evaluate the clinical efficacy of alternate-day administration of S-1 in patients with advanced gastric cancer. The 6-month PFS rate of the alternate-day group was 20.9% and failed to show significant difference from the pre-specified threshold.

Safe and long-term administration of S-1 without impairing quality of life is important for improving the clinical outcomes of gastric cancer. The rationale for alternate-day administration instead of conventional 4-week daily administration is based on the mechanism of action of 5-FU [3]. The cell cycle of normal cells is approximately 12–24 h, the majority of which is S phase. 5-FU, an antimetabolite with high time-dependency, acts in S phase. While many normal cells are not affected by the action of 5-FU by 1-day non-exposure, the cell killing effect of 5-FU would persist for cancer cells that have a longer cell cycle and S phase than normal cells. Based on these facts, Shirasaka et al. developed a method that attenuates gastrointestinal toxicity and bone marrow suppression without decreasing the cancer cell killing effect [12]. A retrospective report showed that alternate-day administration reduced adverse effects and provided a comparable clinical response to that of daily administration [13].

In this randomized, phase II study, patients treated with alternate-day administration of S-1 had a poorer prognosis than those treated with daily administration. The possible reasons for this finding were as follows. There were significant differences in background characteristics between the two groups. Patients assigned to the alternate-day administration group had significantly smaller body surface area and more “Type 4” overall tumor compared to those assigned to the daily administration group. By adjusting for these background factors, apparent different effect on prognosis was not observed. Nevertheless, the most important reason for these differences of prognosis between two groups could have been due to the difference in total dosage of S-1. Patients in the alternate-day administration group had markedly less total dosage of S-1 than the daily administration group. Although, in fact, the tolerability of conventional 4-week daily S-1 administration was found to be better than expected, we have to look into the reasons for the poor dose intensity of the alternate-day S-1 administration in the present study. One possible explanation is the difference of nutritional status of the patient between two groups. Generally, the nutritional status of patients with “Type 4” cancer is poor due to the impairment of dietary intake. The small body surface area of the alternate-day administration group indicated progression of cachexia, resulting in reduction or discontinuation of S-1 chemotherapy. In addition, 55.6% of patients assigned to the alternate-day group were forced to have reduced doses due to renal dysfunction, indicating a possibility that there were more cases of potential impairment of important organs in the alternate-day group than in the daily administration group.

The incidence and time of appearance of adverse events in patients assigned to the alternate-day group was comparable. Another phase II report showed adverse effects in 78% (40/51) of patients, and the incidence of grade 3 and 4 adverse effects was 20% [5]. The major adverse events were decreased hemoglobin, leukopenia, and diarrhea. A phase III report (SPIRITS trial) showed that grade 3 or 4 neutropenia was observed in 40% of patients assigned to S-1 plus cisplatin therapy and 11% of patients assigned to S-1 alone [7]. Grade 3 or 4 anorexia was observed in 30% of patients assigned to S-1 plus cisplatin therapy and 6% of those assigned to S-1 alone. The incidence and pattern of adverse effects in this study were comparable with these studies. There was no obvious evidence that the incidence of severe side effects can be improved in alternate-day groups than daily group.

In conclusion, alternate-day administration of S-1 as the first-line therapy for patients with advanced gastric cancer failed to show significant difference from the pre-specified threshold of PFS, improved tolerability, prolonged OS, or to have a favorable RR. The results of this study suggested that it is important to maintain the administration period and the total dose than the administration method in order to maximize the effect of the S-1 single agent for advanced gastric cancer.
